# Supplementary figures and images for: Infectious disease as a driver of declines and extinctions
Source: Camb Prism Extinct. 2024 Feb 14;2:e2. doi: 10.1017/ext.2024.1 (PMC11895747; doi:10.1017/ext.2024.1)

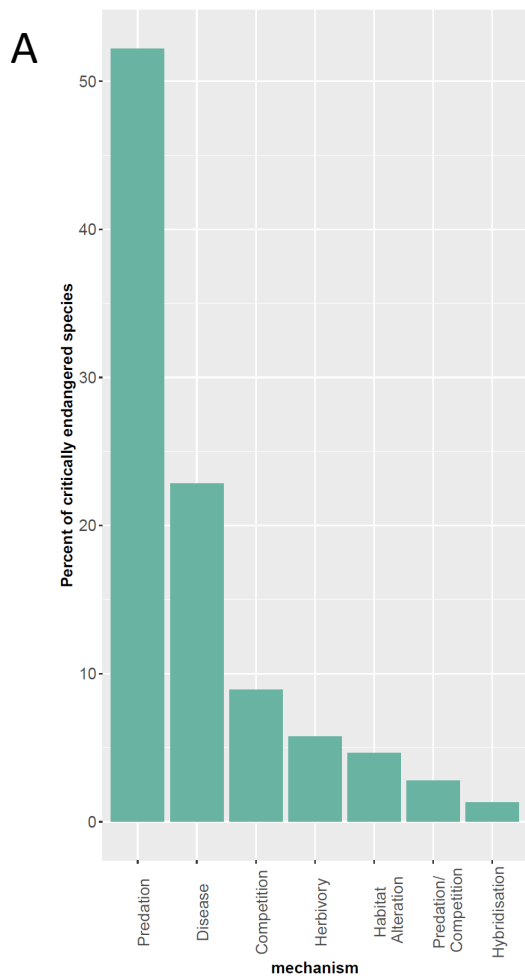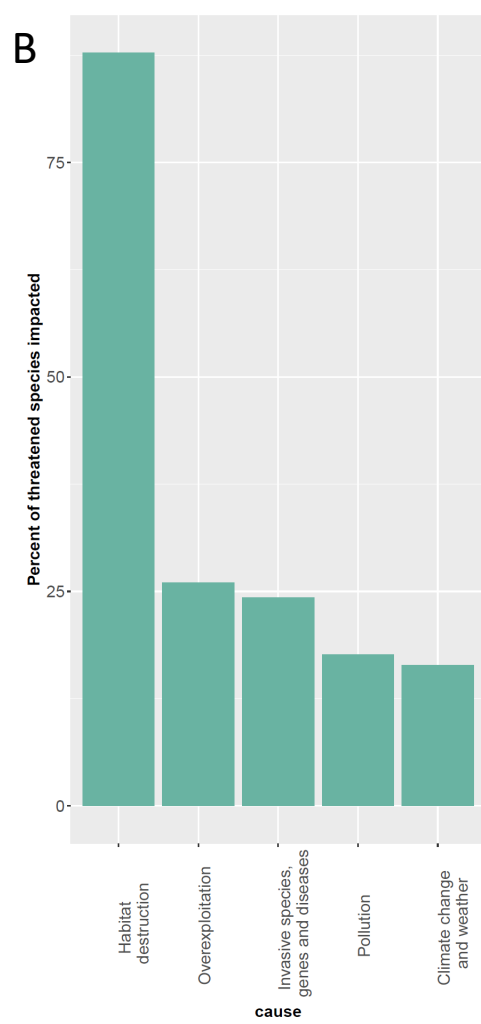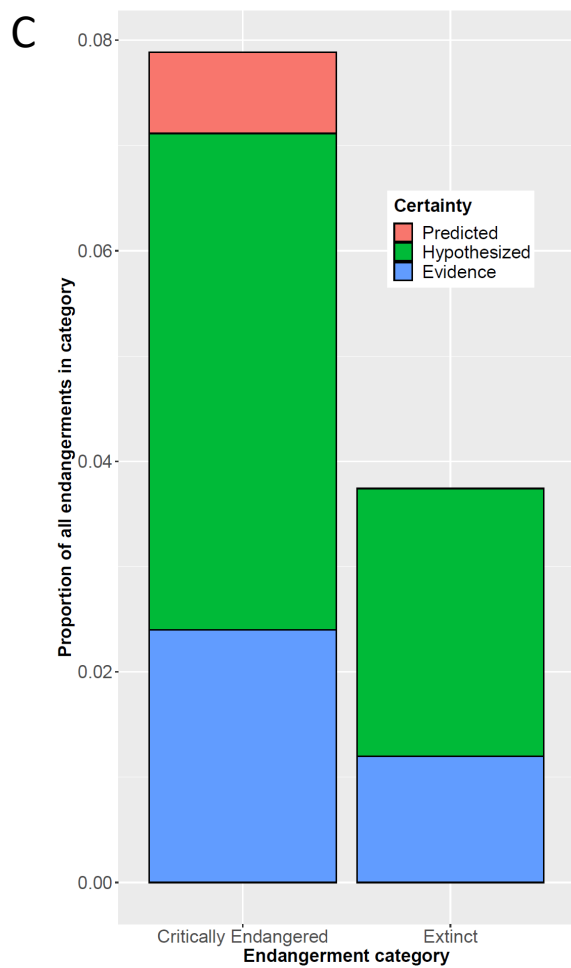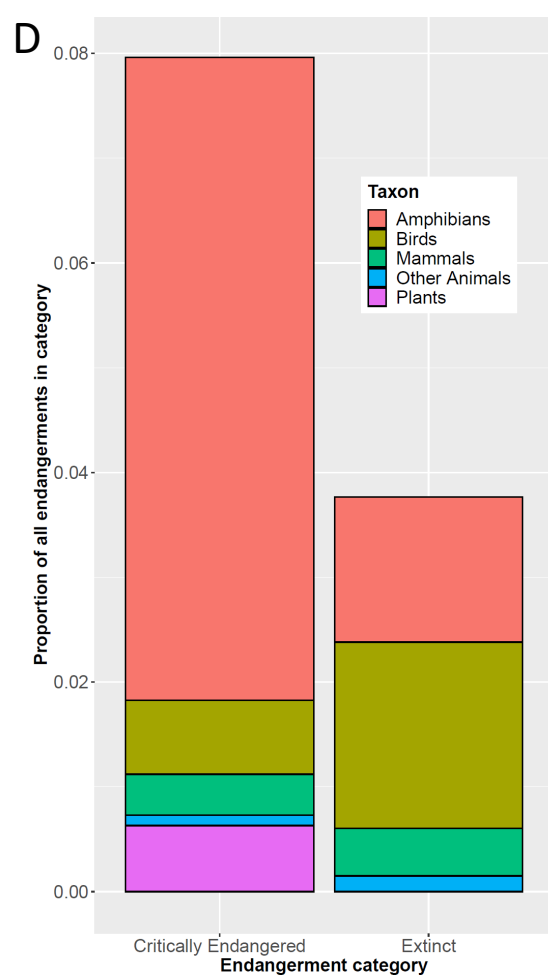

Supplement: McCallum et al. supplementary material 1 — McCallum et al. supplementary material [file S2755095824000019sup001.pdf]
