# Supplementary material for: Infectious disease as a driver of declines and extinctions
Source: Camb Prism Extinct. 2024 Feb 14;2:e2. doi: 10.1017/ext.2024.1 (PMC11895747; doi:10.1017/ext.2024.1)

## Slide 1
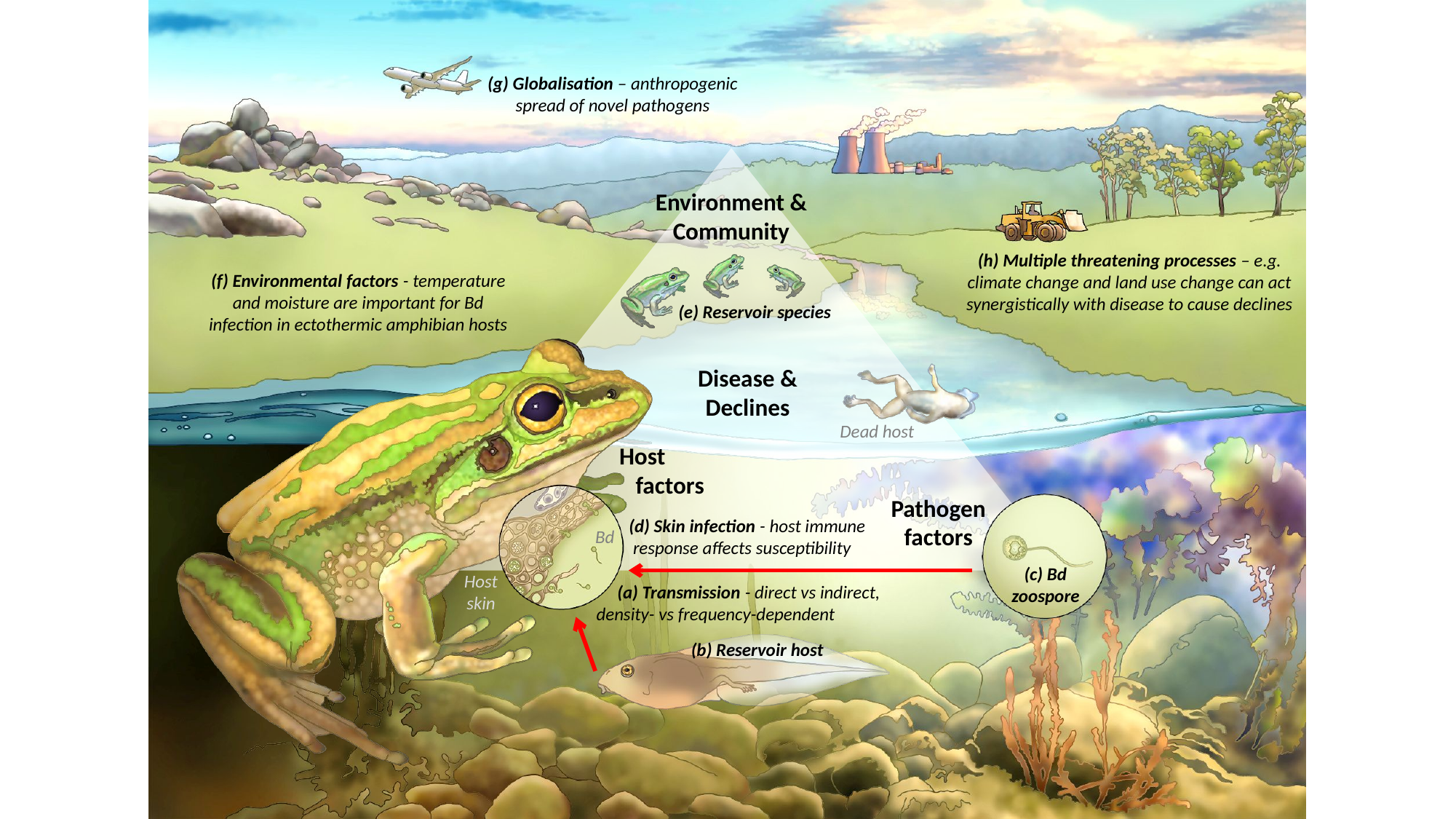

(g) Globalisation – anthropogenic spread of novel pathogens
Environment & Community
(h) Multiple threatening processes – e.g. climate change and land use change can act synergistically with disease to cause declines
(f) Environmental factors - temperature and moisture are important for Bd infection in ectothermic amphibian hosts
 (e) Reservoir species
Disease & Declines
Dead host
Host
 factors
Pathogen factors
(d) Skin infection - host immune
 response affects susceptibility
Bd
(c) Bd zoospore
Host skin
 (a) Transmission - direct vs indirect, density- vs frequency-dependent
(b) Reservoir host

## Slide 2
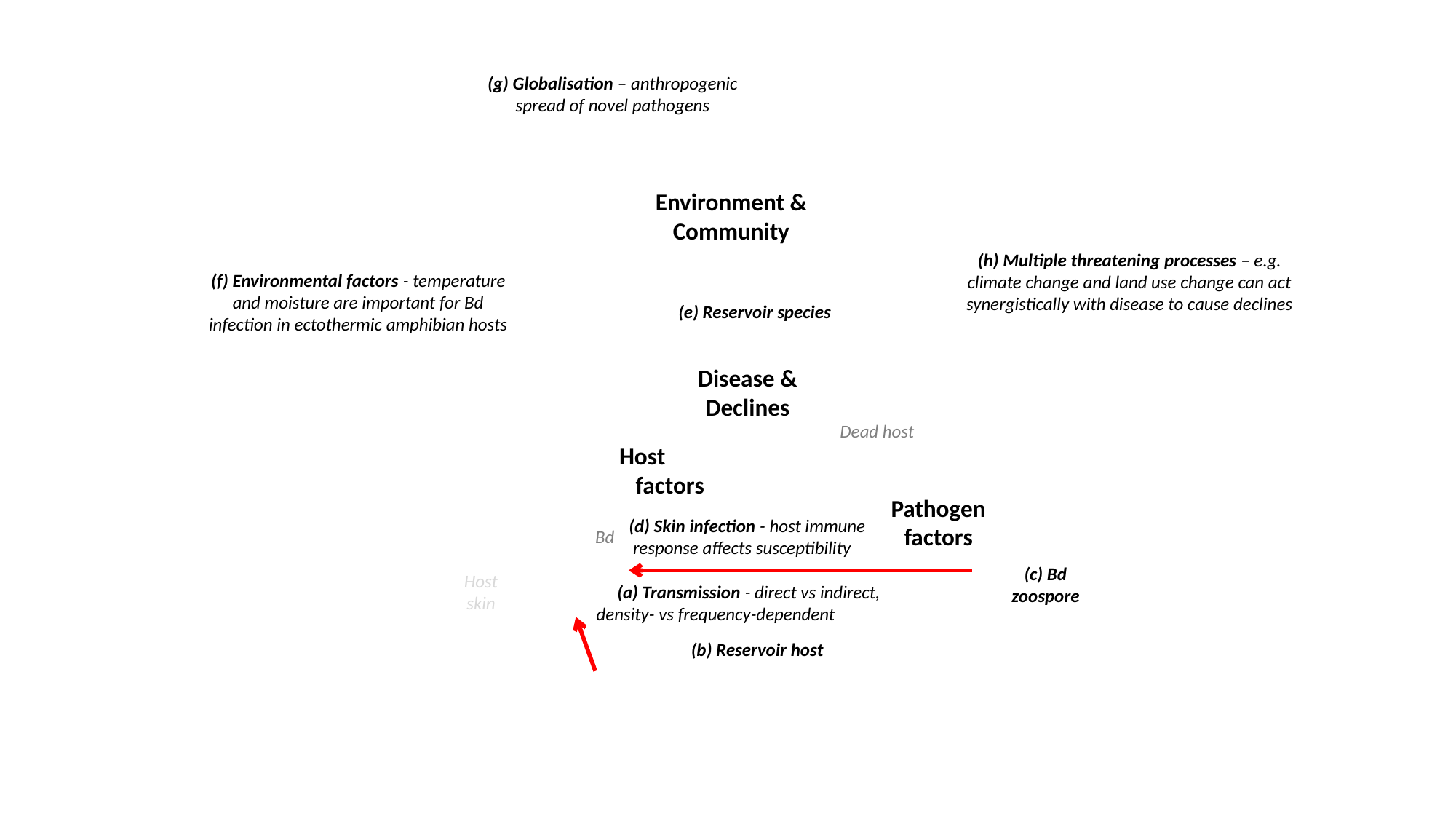

(g) Globalisation – anthropogenic spread of novel pathogens
Environment & Community
(h) Multiple threatening processes – e.g. climate change and land use change can act synergistically with disease to cause declines
(f) Environmental factors - temperature and moisture are important for Bd infection in ectothermic amphibian hosts
 (e) Reservoir species
Disease & Declines
Dead host
Host
 factors
Pathogen factors
(d) Skin infection - host immune
 response affects susceptibility
Bd
(c) Bd zoospore
Host skin
 (a) Transmission - direct vs indirect, density- vs frequency-dependent
(b) Reservoir host

## Slide 3
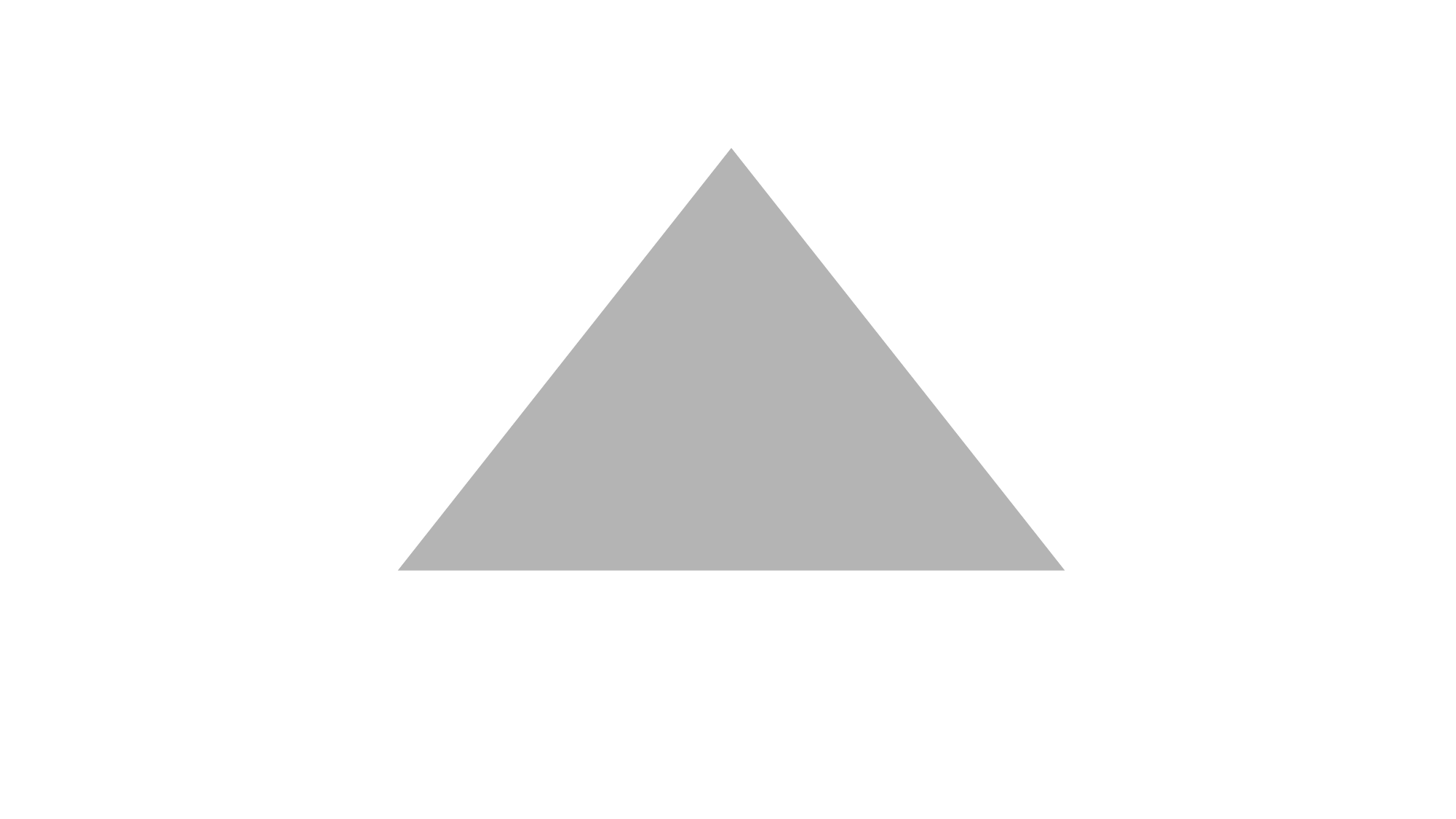

Supplement: McCallum et al. supplementary material 2 — McCallum et al. supplementary material [file S2755095824000019sup002.pptx]
